# Respectful leadership: Reducing performance challenges posed by leader role incongruence and gender dissimilarity

**DOI:** 10.1177/0018726718754992

**Published:** 2018-03-26

**Authors:** Suzanne van Gils, Niels Van Quaquebeke, Jan Borkowski, Daan van Knippenberg

**Affiliations:** Maastricht University, Netherlands, suzanne.vangils@maastrichtuniversity.nl; Kühne Logistics University, Germany, niels.quaquebeke@the-klu.org; Respect Research Group, Germany, jan.borkowski@email.de; Drexel University, USA, dlv39@drexel.edu

**Keywords:** gender dissimilarity, respectful leadership, role congruity theory

## Abstract

We investigate how respectful leadership can help overcome the challenges for follower performance that female leaders face when working (especially with male) followers. First, based on role congruity theory, we illustrate the biases faced by female leaders. Second, based on research on gender (dis-)similarity, we propose that these biases should be particularly pronounced when working with a male follower. Finally, we propose that respectful leadership is most conducive to performance in female leader–male follower dyads compared with all other gender configurations. A multi-source field study (*N* = 214) provides partial support for our hypothesis. While our hypothesized effect was confirmed, respectful leadership seems to be generally effective for female leaders irrespective of follower gender, thus lending greater support in this context to the arguments of role congruity rather than gender dissimilarity.

The perception of female leadership has slowly improved in recent years amidst women’s increasing participation in the workforce (Eagly, 2007). Some studies also hint at a female advantage for certain leadership roles (e.g. Paustian-Underdahl et al., 2014). Nonetheless, both popular surveys (Gallup, 2015; DIW, 2015) and recent research (Koenig et al., 2011; Vial et al., 2016) show that the mental model for leaders still predominantly fits the phrase “think manager–think male” (Schein, 1976; Schein et al., 1996), and women are still falling behind when it comes to top functions (DIW, 2015).

For the increasing amount of female leaders, this mental model of think manager–think male not only translates into tainted perceptions of their leadership quality (e.g. Ayman et al., 2009; Netchaeva et al., 2015; Wang et al., 2013), but also negatively influences the performance of their followers (Tsui et al., 2002; Wang et al., 2013). Much has been written about the challenges female leaders face because of these biased perceptions (Eagly and Carli, 2003). However, a full integrative overview of the relevant factors—follower demographical characteristics, gender differences in the leader–follower dyad, follower performance and the influence of the leadership style—and their theoretical frameworks, is still missing. Against this backdrop, the current article sets out to investigate how female leader–male follower relationships are subject to biases related to the leadership role as well as to gender dissimilarity effects, both of which influence follower performance.

To understand why bias exists against female leaders in the first place, we turn to research on role congruity theory (Eagly and Karau, 2002). This theory argues that when followers are confronted with a female leader, their stereotypes for the leadership role conflict with their stereotypes for the female social role, which often leads to a more negative evaluation of female leaders (Genovese, 1993; Haslam and Ryan, 2008; Koenig et al., 2011; Schein et al., 1996). In turn, followers may develop a poor relationship with the leader and/or perceive her to be less effective, which can have detrimental effects on follower performance (Gerstner and Day, 1997; Ilies et al., 2007). However, only a few recent studies have explored the differences in leadership effectiveness ratings between male and female followers (Ayman et al., 2009; Douglas, 2012; Johnson et al., 2008; Stoker et al., 2012). These studies support the idea that the genders of both leader and follower jointly influence followers’ perceptions of the leader, but they have not yet tested these effects with regard to the effects on follower performance or what type of leadership may be able to overcome the challenges.

While gender dissimilarity may be one issue, we further suggest that the effects on performance can become especially pronounced when female leaders collaborate with male followers. Research on demographical differences between leaders and followers suggests that people find it easier to work with others who are similar to themselves than with those who are demographically different (Lincoln and Miller, 1979; O’Reilly et al., 1989; Tsui and O’Reilly, 1989). Research has shown that followers in demographically different leader–follower dyads are faced with uncertainty, reduced feelings of belonging, reduced job satisfaction and lowered attachment to the organization (Pelled and Xin, 1997; Tsui and O’Reilly, 1989; Tsui et al., 1992; Wesolowski and Mossholder, 1997). Importantly, some studies have argued that the effects of demographic differences can be asymmetrical (Chattopadhyay, 1999). For example, followers generally do not have problems when the leader is older or more educated, but such differences in the other direction conflict with role stereotypes (Chattopadhyay, 1999; Chattopadhyay et al., 2004). Similarly, the effect of gender differences on followers’ ratings of leader effectiveness can depend on the gender of the follower (Chattopadhyay et al., 2004). Although this research on asymmetrical gender effects helps to explain the unique situation that arises for female leader–male follower dyads, these effects are rarely discussed in the context of role congruity. Moreover, many analyses stop at the insight that there are challenges but do not discuss how to overcome them.

We propose that respectful leadership may play a crucial role in reducing the negative effects of gender differences and role incongruity. Respectful leadership is defined as the manifestation of the belief that the other person (i.e. the follower) has dignity and value in his or her own right (Grover, 2013; Van Quaquebeke and Eckloff, 2010). In line with van Knippenberg and Sitkin’s (2013) call to use clearly defined unidimensional aspects of leadership, we argue that investigating respectful leadership as an influencing factor is more appropriate than focusing on multidimensional concepts such as transformational leadership (Bass and Avolio, 1993; Triana et al., 2016), or leader-centric styles such as authentic leadership (Avolio et al., 2009). The foremost reason for our focus on respectful leadership is not only that it is specific, but also is highly relevant to followers feelings of belonging that form the background of our effect, because respectful leadership signals to followers that they are accepted and have status within the team (Grover, 2013; Rogers and Ashforth, 2014; Van Quaquebeke and Eckloff, 2010). Indeed, respectful leadership seems to instill positive feelings about the self (Smith et al., 1998; Van Quaquebeke and Eckloff, 2010), which in turn increases followers’ belongingness and motivation to perform (De Cremer, 2003; De Cremer and van Knippenberg, 2002; Renger and Simon, 2011). These qualities, we will argue, are needed the most when followers are faced with gender differences or role incongruity, and particularly so when both issues appear simultaneously. Building on tenets of respectful leadership research (Van Quaquebeke and Eckloff, 2010), we predict that respectful leadership will have the strongest restorative effect on follower performance when the leader is female and the follower is male.

We tested the hypothesis that for female leader–male follower dyads, who face role incongruity and gender differences, respectful leadership relates particularly positively to follower performance. With the results of our multi-source field study, we add to the literature on role congruity by emphasizing that incongruity effects can possibly be asymmetrical depending on gender differences between the leader and the follower. In addition, by focusing on role congruity, we provide a framework for understanding gender differences that goes beyond mere dissimilarity effects. We explain how the combination of these two effects helps to provide a more fine-grained analysis of the challenges for female leaders, in particular when working with a male follower. We then move beyond the description of the problem by presenting respectful leadership as a potential way to mitigate the negative effect on follower performance.

## Theoretical framework

One challenge for female leaders is that perceptions and assessments of leadership are still strongly influenced by the stereotypically male view of leadership (De Hoogh et al., 2015; Eagly et al., 1995; Paustian-Underdahl et al., 2014). Although some research suggests that there may be a female leadership advantage in some domains (Eagly, 2007; Paustian-Underdahl et al., 2014), a recent meta-analysis assessing leadership stereotypes (Koenig et al., 2011) shows that many people possess a mental model of leadership that is still dominated by traditionally masculine traits (Eagly, 2007). Moreover, workplace dynamics also prohibit those that have a more female-oriented perspective on leadership from expressing it (Hekman et al., 2017). Overall, the traits stereotypically associated with leaders also promote the assumption that men may be more natural leaders (Eagly, 2007; Heilman, 2001). In contrast, recent studies have shown that some feminine traits such as complex interpersonal skills or a communal orientation can also provide women with a leadership advantage in certain leadership roles (Eagly, 2007; Eagly et al., 1995).

Meanwhile, research investigating the differences in the effectiveness of male and female leaders also presents a mixed picture (Van Engen and Willemsen, 2004). Indeed, some studies suggest that followers prefer male leaders overall (Brescoll et al., 2010; Eagly et al., 1992; Elsesser and Lever, 2011), while other studies nuance this view by suggesting that the rater’s characteristics (Ayman et al., 2009; Paustian-Underdahl et al., 2014; Stoker et al., 2012) or the encoding of leadership behaviors (Scott and Brown, 2006) can influence the perception of leader effectiveness. Furthermore, some research attributes the differences in male and female leaders’ effectiveness ratings to women’s personality characteristics, such as a less-potent power motivation (Schuh et al., 2013). In contrast, other studies attribute the differences to leadership perceptions, showing that female leaders are judged more harshly for mistakes than male leaders (Brescoll et al., 2010), that women are often hired for more risky leadership positions than men (Haslam and Ryan, 2008; Rink et al., 2013), and that women need to perform better in order to be promoted (Landau, 1995; Ng et al., 2005), and generally are receiving less respect and admiration (Vial et al., 2016). All in all, the extant research suggests that followers evaluate female leaders differently than their male counterparts. This helps to explain why asymmetrical effects can exist for followers of female leaders compared to followers of male leaders in gender-dissimilar relationships. Building on role congruity theory and research on demographical difference effects, we argue that these asymmetrical effects will in turn affect follower performance.

### Role congruity theory

Positive follower performance depends in part on whether these followers perceive the leader, and by extension the organization, as worthy of commitment (van Knippenberg and Hogg, 2003). According to role congruity theory, the assessment of female leaders’ “worthiness” is influenced by the fact that women’s traditional role expectations contrast with those for leaders, which evokes prejudice against women in leader roles (Eagly and Karau, 2002; Morrison and Von Glinow, 1990). Specifically, the theory posits that a person is perceived as most fitting to a role when the attributes of said role fit the stereotype of the social group the person belongs to (Eagly and Karau, 2002). Role congruity theory is based on the idea that people have a mental image of the attributes or characteristics that are required for certain roles (Hall and Lord, 1995; Lord and Maher, 2002; Sarbin and Allen, 1954). In addition, people hold stereotypes for members of a certain social category, such as gender, as captured in the Social Role Theory (Eagly and Wood, 1991; Koenig and Eagly, 2014). Social role expectations for the female gender traditionally describe women as communal and warm (Fiske et al., 2002). In contrast, as discussed above, the attributes associated with the leadership role are often more masculine and agentic (Koenig et al., 2011; Schein et al., 1996; Sczesny, 2003). Thus, when people compare their mental images of men and leaders, they are comparing highly congruent and redundant information, while the mental comparison of women and leaders generates incongruent and diverging information (Eagly and Karau, 2002).

Role congruity theory thus helps to explain why prejudice against female leaders arises irrespective of the follower’s gender (Eagly and Karau, 2002). To resolve the discrepancy between their mental images for women and leaders, observers will (often implicitly) conclude that the woman is either not a good leader—because she violates the norms and attributes associated with the leadership role (Schein et al., 1996)—or is less warm and communal than the average woman and thus a bit like an “Iron lady” (Genovese, 1993). Role congruity theory argues this leads to a general (and implicit) bias that a female leader is less suitable for the job (Eagly and Karau, 2002; Haslam and Ryan, 2008). Consequently, female leaders are perceived as less legitimate, and therefore evoke less admiration and respect from their followers (Vial et al., 2016). As a result of such poor assessment, followers may (often implicitly) conclude that a female leader is less worthy to commit to and thus be less willing to contribute to the organization in terms of performance. Indeed, reduced performance can be expected simply as a function of friction in the leader–follower relationship. Additionally, social identity theorizing on leadership (van Knippenberg and Hogg, 2003) argues that followers perceive leaders as signposts of the organization. Therefore their relationship with the leader will inform their relationship to the organization as a whole, and as such extends to followers’ willingness to perform for the organization.

### Leader gender and similarity

However influential, research on role incongruity does not tell the entire story of how biases influence the perceptions of female leaders. Recent studies on the evaluation of female leaders have started to disentangle the idea that female leaders are generally evaluated more negatively, and instead posit that rater characteristics play an important role (Paustian-Underdahl et al., 2014; Powell and Butterfield, 2015; Sczesny, 2003). Indeed, male followers seem to be more skeptical of female leaders than female followers. For example, males have been found to provide lower ratings of female transformational leadership than their female counterparts (Ayman et al., 2009), and feel more threatened by female leaders (Netchaeva et al., 2015), although these ratings arguably improve when female leaders mix their feminine style with masculine behaviors (Kark et al., 2012). Also, female narcissistic leaders were rated as less effective than male narcissistic leaders, but this difference was only found for male followers (De Hoogh et al., 2015). Other studies find that female followers prefer a more (typically feminine) considerate leadership style (Vecchio and Boatwright, 2002), and by extension, female leaders in general (Stoker et al., 2012). Together these studies suggest that the negative evaluation of female leaders may be stronger for male followers.

The different reactions of female and male followers to female leaders can be partly explained by research on leader–follower similarity. Research on the similarity-attraction paradigm (Byrne, 1971), suggests that liking increases when others are visibly similar or have similar values. Based on this, it is suggested that similar dyads perform better because they profit from the effects of interpersonal attraction. In addition, social categorization theory (Turner et al., 1987) suggests that physical (as well as social and status-based) similarity helps individuals to make inferences about the similarity between their own and others’ attitudes, thereby offering a sense of belonging. Naturally, dissimilar leader–follower dyads do not profit from the positive effects of similarity. Rather, research showed that dissimilarity increased followers’ role ambiguity and decreased leader–follower affect (Tsui and O’Reilly, 1989; Tsui et al., 1992). Research on leadership shows that leaders of the opposite gender are preferred less and rated as less effective than leaders of the same gender, regardless of the gender composition of the dyad (see Powell and Butterfield, 2015; Stoker et al., 2012). In addition, leaders of the opposite gender can negatively affect the relationship between empowerment and performance (Avery et al., 2013), or collaboration and performance in general (Tsui et al., 2002). For this reason, leader–follower gender similarity may make female followers of a female leader feel that they belong to the leader and in extension to the organization. In contrast, the resultant sense of lower belonging may evoke more negative responses in male followers toward their female leader (Netchaeva et al., 2015). Following our arguments above, we suggest that these mechanisms (e.g. van Knippenberg and Hogg, 2003) will lead to impaired follower performance in male versus female followers of female leaders.

Integrating research on similarity and role congruity, we thus expect that the effects of role congruity on follower performance are especially strong in leader–follower dyads in which the leader is female and the follower is male. Specifically, male followers are not only faced with a role-incongruent leader, but also do not profit from gender similarity effects. Male followers may thus experience two challenges at once when working with a female leader, both of which impair their relationship with the leader. Consequentially, we argue, this translates into poorer performance. In contrast, female followers only experience one challenge at a time: either they are faced with a female leader, who is role incongruent but similar in gender, or they are faced with a male leader, who is role congruent but dissimilar in gender:

*Hypothesis 1*: The dyadic gender composition influences performance, such that performance in dyads in which the leader is female and the follower is male is lower than in dyads of any other composition.

### Respectful leadership as a buffer for detrimental relational demographic effects

Interestingly, although many of the above-cited studies focus on leaders’ gender, they do not address the issue of how leaders may address the challenges associated with gender and leadership through leader behavior. This is remarkable because leadership behaviors are often key to resolving conflict situations in teams or overcoming strained leader-follower relationships (Giessner et al., 2009). Framed the other way around, research found that leadership behavior becomes more influential when there is increased insecurity based on dissimilarity, because this insecurity leads followers to assess their leader’s behavior with greater scrutiny (Duffy and Ferrier, 2003). Related, a recent study, for instance, has also shown that when female leaders show administrative rather than ambitious agency, the threat experienced by male followers is reduced (Netchaeva et al., 2015). Indeed, confrontations with role incongruence and gender differences can provide leaders with an increased opportunity to influence followers, and restore their sense of belonging, because more critical incidents occur.

Although some have argued that this may be accomplished when female leaders display a mixture of masculine and feminine behavior (Kark et al., 2012), we argue that a more general follower focused leadership style may be more appropriate. Based on this rationale, we focused on respectful leadership, a unidimensional leadership construct that is specifically tailored to these demands (see van Knippenberg and Sitkin, 2013). In contrast to styles that mainly focus on the leader and their expression of their style (such as ethical leadership or authentic leadership; see Avolio et al., 2009), respectful leadership is inherently focused on recognizing the follower as a valuable person in the organization in their own right (Van Quaquebeke and Eckloff, 2010). Theoretical considerations of respect suggest that its beneficial effect becomes especially cogent in critical situations involving conflict (-potential) (Dillon, 2007; Grover, 2013). In this study, we propose that respectful leadership is the right style for overcoming challenges that are owing to role incongruence or gender differences in leader–follower dyads, because of its potential buffering effect in situations where a female leader is paired with male followers.

Respectful leadership is built on the idea that followers are self-reliant people worthy of fair and supportive treatment (Clarke, 2011; Clarke and Mahadi, 2017; Decker and Van Quaquebeke, 2015; Grover, 2013), who should be treated with dignity and be appreciated for their work contributions (Grover, 2013; Van Quaquebeke and Eckloff, 2010). Leaders’ enactment of such positive treatment signals to followers that they have status and are accepted in the organization (Huo and Binning, 2008; Huo et al., 2010); it also increases their feelings of autonomy, relatedness and competence (Decker and Van Quaquebeke, 2015; Van Quaquebeke and Eckloff, 2010), as well as their self-esteem (De Cremer and Blader, 2006; Renger and Simon, 2011; Smith et al., 1998). In sum, respect has proved to have an important social function, as it provides insight into one’s acceptance and status within the group (Tyler, 2001; Tyler and Smith, 1999), which can then translate into improved organizational commitment (Sluss and Ashforth, 2008) and job satisfaction (Decker and Van Quaquebeke, 2015). In the current article, we extend these implications to the domain of follower performance.

In short, we suggest that leaders who employ respectful leadership strategies can alleviate follower concerns about not fitting to the leader and by extension the organization. As discussed above, these concerns could stem from gender dissimilarity and role incongruence, and reduce follower performance. Respectful leadership ensures that the follower feels valued as a person (Van Quaquebeke and Eckloff, 2010), and establishes an interaction that goes beyond mere role expectations and dissimilarities, which in turn makes the follower experience increased belonging to the organization. As such, we suggest that respectful leadership should be particularly effective in addressing the asymmetrical performance effects of leader–follower gender dissimilarity and role incongruity in female leader–male follower dyads. As we expect this effect as a result of the combination of all three factors simultaneously, we summarize our prediction in a three-way hypothesis:

*Hypothesis 2*: The effect of dyadic gender composition on performance is moderated by respectful leadership, such that performance in dyads in which the leader is female and the follower is male is more strongly improved by respectful leadership, than in dyads of any other composition.

## Method

### Data and sample

The participants in this study included 214 followers and their respective 214 leaders from 10 German organizations. Following announcements about the study by senior management, participants either filled out the questionnaire online or in pencil-and-paper format. For each leader–follower dyad, followers provided ratings of respectful leadership, while leaders provided ratings of follower performance. Followers were on average 39 years old (SD = 11.80) and 59% were female. Their tenure with the company ranged from less than 1 to 35 years (*M* = 7.94, SD = 7.85). Leaders were on average 45 years old (SD = 9.47) and 42% were female. Leaders’ tenure with the company ranged from less than 1 to 35 years (*M* = 11.83, SD = 8.47). Of the leader–follower dyads, 30% worked in a government agency, 25% worked in social services, 18% worked in technology, and the other 27% worked in other types of industries. As expected, the descriptive statistics show that, on average, leaders in our sample were older and had longer tenure than followers. Leaders in our sample were more often male (58%), whereas a higher percentage of followers were female (59%). This resulted in 143 same-gender dyads (69 male–male dyads, 32% of total; 74 female–female dyads, 35% of total) and 71 different-gender dyads (54 male leader-female follower dyads, 25% of total; 17 female leader–male follower dyads,^1^ 8% of total). In exchange for their participation, which was voluntary, participants were entered into a lottery for book vouchers.

### Measures

#### Gender

Both leaders and followers were coded as 1 = male, 0 = female. We computed a variable denoting gender similarity in which a score of 1 indicated gender dissimilarity and a score of 0 indicated gender similarity.

#### Follower performance

Follower performance was rated by the leader of each follower on a four-item performance quality scale (Ashford et al., 1989). An example item is ‘he/she delivers work of high quality’ (1 = *disagree completely*, 5 = *agree completely*).

#### Respectful leadership

Respectful leadership was rated by the followers using Van Quaquebeke and Eckloff’s (2010) 12-item respectful leadership scale. Example items are ‘my leader shows a genuine interest in my opinions and assessments’ and ‘my leader takes me and my work seriously’ (1 = *disagree completely*, 5 = *agree completely*).

#### Control variable

Frequent communication may make the follower aware of the leader’s respect toward them, and thus increase the effect of respectful leadership (Clarke and Mahadi, 2017; Giessner and Van Quaquebeke, 2010). Moreover, communication intensity correlated strongly with performance and respectful leadership in our model and could theoretically be an alternative explanation for our findings. Thus, we included it as a control variable in our subsequent analyses (Spector and Brannick, 2011). It was rated by followers on a four-item scale (McAllister, 1995). An example item is ‘how often do you have work-related encounters with your leader?’ All items were rated on a scale ranging from 1 (*once or twice in the past 6 months*) to 7 (*multiple times per day*), α = .86. The mean for this scale was 4.37 (SD = 1.51)—which indicates that participants had on average 1 or 2 (scale point 4), or multiple interactions (scale point 5) with their leader per week. Seven participants did not supply answers for this variable and were thus removed from the sample. Removing these participants from the analyses did not affect the results.

## Results

Before we started the hypotheses testing, we inspected the relative amount of variance at the individual and the organization level. For the organization-level intra-class correlation coefficient (ICC)1 for performance was .12, ICC2 was .81. ICC1 for the team level was .38, and ICC2 was .66. Based on this, we decided to analyze the data as a three-level multilevel model, with followers nested into teams nested into organizations. The results for a fixed slope model with followers nested in teams in organizations are presented below. We did not test random slope models, as an unconditional random coefficient model showed insufficient variance at the team level, γ_10_ = .06, Wald Z = 0.60, *p* = .546.

Table 1 provides an overview of the demographics by gender pairing. Table 2 presents Cronbach’s alpha and inter-correlations between the key variables in our study.

**Table 1.** Sample demographics.

	Fem L–Male F	Male L–Fem F	Fem L–Fem F	Male L–Male F
Follower performance	3.78 (0.96)^a^	4.38 (0.75)^b^	4.28 (0.84)^b^	3.95 (0.76)^a^
Respectful leadership	4.32 (0.97)	4.34 (0.72)	4.45 (0.66)	4.24 (0.73)
Communication	3.85 (1.18)	4.77 (1.71)	4.22 (1.43)	4.35 (1.59)
Leader age	49.06 (6.45)	47.30 (7.99)	45.77 (10.09)	43.81 (9.50)
Follower age	42.71 (13.72)	40.80 (10.44)	37.43 (11.52)	39.52 (12.72)
*N*	17	54	74	69

L = leader; F = follower; *N* = 214, ^*^p < .05, ^**^p < .01. Performance ratings with a different superscript differ significantly.

**Table 2.** Inter-correlations.

	1	2	3	4	5	6
1. Follower performance	(.88)					
2. Respectful leadership	.31**	(.94)				
3. Communication	.24**	.25**	(.84)			
4. Leader gender	.06	.06	−.12			
5. Follower gender	.27**	.08	.06	.37**		
6. Leader age	.16*	.14*	−.07	.06	.08	
7. Follower age	−.02	.22**	−.05	−.07	−.05	.33**

*N* = 214, ^*^p < .05, ^**^p < .01. Relevant Cronbach’s alpha’s are listed between brackets on the diagonal.

### Regression results

**Figure 1.**
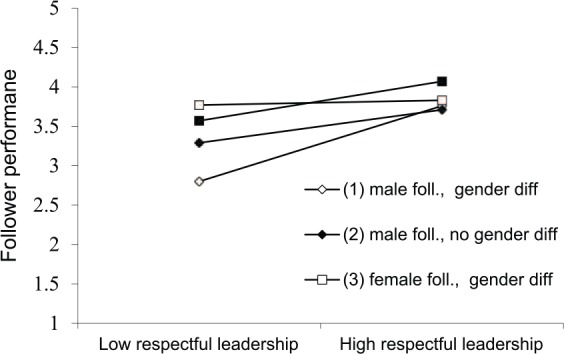
Simplified plot of the three-way interaction between respectful leadership, follower gender and gender difference.

Multi-level analysis results are reported in stepwise order in Table 3. Close inspection of Table 3, Model 2, shows that there was a significant positive main effect for respectful leadership on performance, *B* = .24, *SE* = .05, *t*(210.70) = 4.52, *p* = .000, 95% Confidence Interval (CI;.14;.34), and a negative significant effect of gender on performance, *B* = −.40, *SE* = .11, *t*(216.24) = −3.55, *p* = .000, *95% CI*(−.61;.−.18), suggesting that the female followers in our sample and those with a more respectful leader performed better on average. As can be observed in Model 3, none of the two-way interactions were significant. Thus, Hypothesis 1, that male followers with a female leader would perform worse was not confirmed, *B* = −.15, *SE* = .32, *t*(75.67) = −0.47, *p* = .639, *95% CI*(−.79;.49). Inspection of the three-way interaction confirmed the proposed effect, *B* = .49, *SE* = .22, *t*(200.39) = 2.18, *p* = .031, *95% CI*(.05;.93). Respectful leadership positively influenced follower performance in dyads with gender differences, in which the leader was female and the follower was male, confirming Hypothesis 2. This interaction is depicted in Figure 1 (respectful leadership is depicted on the x-axis to facilitate interpretation).

**Table 3.** Multilevel regression results of the predicted three-way interaction between follower gender, gender dissimilarity and respectful leadership, with and without controlling for communication intensity—followers nested into teams into organizations.

	Model 1			Model 2			Model 3			Model 4		
	B	SE	*p*	B	SE	*p*	B	SE	*p*	B	SE	*p*
Communication	.12**	.03	.000	.08*	.03	.011	.08*	.03	.016	.09**	.03	.008
Follower gender (FG)				−.37**	.11	.001	−.34*	.17	.046	−.32	.17	.056
Gender dissimilarity (GD)				−.09	.11	.438	−.04	.17	.806	−.02	.17	.925
Respectful leadership (RL)				.22**	.05	.000	.15	.09	.098	.25*	.10	.012
FG × Gender dissimilarity							−.15	.32	.639	−.20	.32	.542
FG × Respectful leadership							.15	.10	.157	−.04	.12	.787
GD × RL							.01	.11	.946	−.22	.14	.142
FG × GD × RL										.49*	.22	.031
Variance components												
Within follower variance	.24**	.07		.21**	.06		.21**	.06		.21**	.06	
Residual variance	.41**	.05		.36**	.04		.36**	.04		.35**	.04	
−2 Restricted log likelihood		485.81			467.20			470.72			467.18	

SE = standard error. *N* = 214, ^*^p < .05, ^**^p < .01.

To analyze the slope of respectful leadership for each dyadic composition, we multiplied the variable respectful leadership with a dummy variable, coded 1 for the designated dyadic composition and 0 for all other compositions (West et al., 1996). This analysis showed that respectful leadership had the strongest positive effect on female leader–male follower dyads, *B* = 0.42, *SE* = .14, *t*(185.17) = 3.08, *p* = .002, 95% CI (.15; .70). However, the effect of respectful leadership on female leader–female follower dyads was significant as well, *B* = 0.31, *SE* = .10, *t*(205.09) = 3.05, *p* = .003, as was the effect on male leader–male follower dyads, *B* = 0.21, *SE* = .09, *t*(179.58) = 2.36, *p* = .019. The effect of respectful leadership was not significant on male leader–female follower dyads, *B* = 0.04, *SE* = .11, *t*(207.96) = 0.39, *ns*. Following Dawson and Richter’s (2006) approach, we additionally conducted slope difference tests, which showed that the slope for female leader–male follower and male leader–female follower dyads differed significantly, *t*(212) = 2.60, *p* = .010, and the slope for female leader–male follower and male leader–male follower dyads differed marginally, *t*(212) = 1.71, *p* = .089. Unexpectedly, none of the other slopes differed from each other.

## Discussion

Our findings partially confirm the hypotheses. Hypothesis 1 is not confirmed as we found a lower performance for male followers overall, rather than a specific effect of male followers of female leaders. Hypothesis 2 was partially confirmed, however; the results of the three-way interaction showed that respectful leadership was positively related to performance in dyads with a female leader and a male follower. This effect differed from the relationship between respectful leadership and performance in dyads consisting of male leaders with either a female (significant) or a male (marginally) follower, but, contrary to our expectations, not from the effect for dyads consisting of a female leader and a female follower. These findings partially substantiate our argument that respectful leadership may be particularly effective for performance in those dyads that face role incongruity on the part of the leader, alongside gender differences between leaders and followers. Controlling for communication between leaders and followers did not change the results, which highlights that the effect lies in the content, rather than the frequency, of communication.

### Theoretical implications

Our findings provide a more extended explanation for why gender dissimilarity is a larger problem for female leader–male follower dyads than for male leader–female follower dyads, and why respect may be particularly effective to address the problems of uncertainty and decreased belongingness in this case. Interestingly, we did find effects of respectful leadership for gender similar dyads as well. For the female leader–female follower dyads, this can be explained by the ‘Queen Bee’ phenomenon, the effect that senior women compete with junior women in the organization, thereby hindering their career progress (Cooper, 2011; Derks et al., 2016). This Queen Bee behavior can be equated with a lack of respectful leadership, which has been related to negative follower outcomes (Decker and Van Quaquebeke, 2015). For male–male dyads the effect may be explained by status competition and rivalry that exist at low levels of respect. To this end, our study produces a practical insight: although leaders cannot resolve the gender dissimilarity or perceived role-incongruence, they can improve the reduced performance resulting from it.

We aimed to show that integrating the literature on role congruity theory (Eagly and Karau, 2002) and the literature on relational gender differences (Douglas, 2012; Johnson et al., 2008) can explain the presence of negative effects in female leader–male follower dyads, especially when these effects occur simultaneously. Our findings extend the literature on role congruity theory (Eagly and Karau, 2002) by emphasizing that the follower’s gender is important to understanding the effects of role incongruity, as it helps to differentiate how it affects female leadership.

Moreover, research in both the domain of role incongruity or the domain of gender differences has mainly focused on describing the negative consequences of role incongruity or gender differences for the leader–follower relationship (Douglas, 2012; Scott and Brown, 2006; Sczesny, 2003) or leader evaluations (Paustian-Underdahl et al., 2014). We extend these lines of research to the concept of performance. Indeed, there are remarkably few studies that investigate the effect of either leader role incongruity (Wang et al., 2013), or gender differences (Tsui et al., 2002), on actual follower performance, despite the amount of literature discussing gender differences in leadership (Eagly, 2005; Eagly et al., 1995).

Earlier research has shown that surface-level dissimilarities can be overcome once deeper-level similarities are discovered (Guillaume et al., 2012). Our research extends this idea by suggesting that respect might particularly help followers of female leaders to reconsider their initially experienced uncertainty and move toward deeper-level considerations. Relatedly, research on identification in organizations has shown that when followers identify more strongly with their leader, their support for the leader becomes less dependent on the leader’s stereotypicality (Hogg et al., 1998). Respectful leadership can help increase identification (Van Quaquebeke and Eckloff, 2010) by making followers feel valued despite the dissimilarity with their leader. Following this logic, it could be that respectful leadership accelerates the discovery of deep-level similarities, restores follower feelings of belonging, and thereby reduces the effects of gender dissimilarity and role incongruity. Future research should explore whether this also motivates reciprocation of respect toward the leader by the follower.

Although various researchers have investigated the positive effects of respect on followers and have argued that its full potential likely only unfolds under condition of conflict (De Cremer, 2003; Renger and Simon, 2011; Simon et al., 2006; Van Quaquebeke and Eckloff, 2010), our research is, to our knowledge, the first to introduce respectful leadership as a moderator that can attenuate a difficult situation. As such, our research also contributes to the literature on respectful leadership. Respectful leadership is specifically aimed at accepting and understanding the follower as a person of inherent value (Grover, 2013; Van Quaquebeke and Eckloff, 2010), and may thus be the most fitting leadership style in this context. A recent study showing that female leaders are often less admired and respected (Vial et al., 2016), may emphasize that respect is a crucial factor in this case even more. Although our conclusions here revolve around the effectiveness of respectful leadership, this does not imply respectful leadership is the only leadership style that can overcome the challenges associated with gender dissimilarity.

Importantly, while our research has focused on gender differences, other studies have shown similar asymmetrical effects related to other demographic differences (Guillaume et al., 2012; Tsui et al., 2002), such as occupational status (DiBenigno and Kellogg, 2014; Phillips et al., 2009), perceived mentoring (Lankau et al., 2005; Turban et al., 2002), job satisfaction, belonging and attachment (Pelled and Xin, 1997; Tsui et al., 1992; Wesolowski and Mossholder, 1997), and ultimately lower performance (Luksyte and Avery, 2015; Tsui et al., 2002). In addition, despite the fact that our German sample should be relatively similar to other western samples on the crucial cultural dimensions (e.g. powerdistance, masculinity or individualism; Hofstede, 1983), some cultural specifics may exist in our sample (Brodbeck et al., 2002). Addressing the moderation of respectful leadership for the relationship of each of these cultural factors, or combinations of these factors, and performance seems a fruitful avenue for future research that was, however, beyond the scope of what we could investigate with our present sample.

### Managerial implications

In the move toward greater workforce diversity, organizations have to deal with the fact that diversity necessarily invites dissimilarity, which can pose challenges to collaboration. Our research is among the first to suggest a solution—namely, respectful leadership—to overcome the challenges affecting female leaders in a negative way. Research has shown that respect is one of the most valued aspects in leadership (Van Quaquebeke et al., 2009). Crucial components of respectful leadership, such as social skills and social awareness in leaders (Day, 2000; Pearce, 2007) as well as respectful communication by means of question-asking style, could be easily implemented in leadership trainings. When female leaders act as a respectful role model and make the follower feel valued (see Van Quaquebeke and Eckloff, 2010), followers may reciprocate the respect, alongside increased belonging and restored follower performance.

### Strengths, limitations and suggestions for future research

The presented research is based on multi-source multilevel data in which followers rated the respect they experienced from their leader and leaders rated the followers’ performance. This approach eliminates same-source bias (Podsakoff et al., 2003) and thus ensures that perceptual biases associated with single-source data did not influence the results. Although biases at the dyadic level may exist (Strauss et al., 2001), such biases are unlikely to adversely influence or explain the interactional effects in our study, and for this reason we consider our multi-source approach to be a strength of our research. In addition, the fact that we did not find an interaction effect of gender and dissimilarity, or for dissimilarity in itself, speaks against a potential dissimilarity bias. Nonetheless, future research could employ experimental methods, in which leaders rate unknown followers, to rule out the potential biases with certainty.

One potential limitation of our study is the unequal sample size between gender-dissimilar dyads. In our sample, there was a particularly small amount of dyads with a female leader. Fortunately, these dyads were evenly distributed across the organizations participating in our study, and there were no differences in terms of the distribution or variance of this group compared to the dyads of other compositions. Our sample size in general, and this group specifically, is somewhat limited, and thus the power of this study might be limited as well. However, this distribution of the dyads also represents a structural difference in the population with regard to the amount of women in leader roles (see Eagly and Karau, 2002). In addition to reasons for the lower amount of female leaders described elsewhere (Eagly and Karau, 2002; Heilman, 2001), it might be that the low amount of female leader–male follower dyads can be explained by the mechanisms described in this article. These effects might lead to a higher level of turnover or layoffs in these dyads, or biases in the initial structuring of teams. However, it is important to emphasize that despite this limitation, our research is based on reports from actual leader-follower dyads rather than simulated situations through vignettes or questions about ideal leader stereotypes (Elsesser and Lever, 2011). Nonetheless, future research should explore our findings in the context of different dependent variables, such as turnover and general job satisfaction, to discover whether leader–follower dyads composed of different genders are more likely to dissolve.

In conclusion, our study contributes to this by suggesting respectful leadership as a style that leaders can use to their advantage when role incongruence or gender differences disadvantage them. This approach could, in combination with systemic approaches like fostering a diversity climate, eventually habituate especially male followers to female leaders. Ultimately, mutual respect will help to shift the responsibility for overcoming the ‘think manager–think male’ stereotype from the female leaders to all members of the organization.

## Notes

1 These dyads were located in four of the 10 organizations in the sample, distributed over 10 teams. Included industries were healthcare, general sales, consultancy and logistics. Industries present in the other teams but not in the female leader–male follower dyads are sales and services, childcare and IT (*N* =1). Based on this, we do not see a reason to suspect a bias based on industry.

## References

[bibr1-0018726718754992] Ashford SJ Lee C Bobko P (1989) Content, causes, and consequences of job insecurity: A theory-based measure and substantive test. Academy of Management Journal 32(4): 803–829.

[bibr2-0018726718754992] Avery DR Wang M Volpone SD Zhou L (2013) Different strokes for different folks: The impact of sex dissimilarity in the empowerment-performance relationship. Personnel Psychology 66(3): 757–784.

[bibr3-0018726718754992] Avolio BJ Walumbwa FO Weber TJ (2009) Leadership: Current theories, research, and future directions. Annual Review of Psychology 60: 421–449. 10.1146/annurev.psych.60.110707.163621 18651820

[bibr4-0018726718754992] Ayman R Korabik K Morris S (2009) Is transformational leadership always perceived as effective? Male subordinates’ devaluation of female transformational leaders. Journal of Applied Social Psychology 39(4): 852–879.

[bibr5-0018726718754992] Bass BM Avolio BJ (1993) Transformational leadership and organizational culture. Public Administration Quarterly 17(1): 112–121.

[bibr6-0018726718754992] Brescoll VL Dawson E Uhlmann EL (2010) Hard won and easily lost: The fragile status of leaders in gender-stereotype-incongruent occupations. Psychological Science 21(11): 1640–1642. 20876882 10.1177/0956797610384744

[bibr7-0018726718754992] Brodbeck FC Frese M Javidan M (2002) Leadership made in Germany: Low on compassion, high on performance. Academy of Management Executive 16(1): 16–29.

[bibr8-0018726718754992] Byrne DE (1971) The Attraction Paradigm. New York: Academic Press.

[bibr9-0018726718754992] Chattopadhyay P (1999) Beyond direct and symmetrical effects: The influence of demographic dissimilarity on organizational citizenship behavior. Academy of Management Journal 42(3): 273–287.

[bibr10-0018726718754992] Chattopadhyay P George E Lawrence SA (2004) Why does dissimilarity matter? Exploring self-categorization, self-enhancement, and uncertainty reduction. Journal of Applied Psychology 89(5): 892–900. 15506868 10.1037/0021-9010.89.5.892

[bibr11-0018726718754992] Clarke N (2011) An integrated conceptual model of respect in leadership. Leadership Quarterly 22(2): 316–327.

[bibr12-0018726718754992] Clarke N Mahadi N (2017) Mutual recognition respect between leaders and followers: Its relationship to follower job performance and well-being. Journal of Business Ethics 141(1): 163–178.

[bibr13-0018726718754992] Cooper VW (2011) Homophily or the Queen Bee syndrome: Female evaluation of female leadership. Small Group Research 28(4): 483–499.

[bibr14-0018726718754992] Dawson JF Richter AW (2006) Probing three-way interactions in moderated multiple regression: Development and application of a slope difference test. Journal of Applied Psychology 91(4): 917–926. 16834514 10.1037/0021-9010.91.4.917

[bibr15-0018726718754992] Day DV (2000) Leadership development: A review in context. Leadership Quarterly 11(4): 581–613.

[bibr16-0018726718754992] De Cremer D (2003) Noneconomic motives predicting cooperation in public good dilemmas: The effect of received respect on contributions. Social Justice Research 16(4): 367–377.

[bibr17-0018726718754992] De Cremer D Blader SL (2006) Why do people care about procedural fairness? The importance of belongingness in responding and attending to procedures. European Journal of Social Psychology 36(2): 211–228.

[bibr18-0018726718754992] De Cremer D van Knippenberg D (2002) How do leaders promote cooperation? The effects of charisma and procedural fairness. Journal of Applied Psychology 87(5): 858–866. 12395810 10.1037/0021-9010.87.5.858

[bibr19-0018726718754992] De Hoogh AHB Den Hartog DN Nevicka B (2015) Gender differences in the perceived effectiveness of narcissistic leaders. Applied Psychology 64(3): 473–498.

[bibr20-0018726718754992] Decker C Van Quaquebeke N (2015) Getting respect from a boss you respect: How different types of respect interact to explain subordinates’ job satisfaction as mediated by self-determination. Journal of Business Ethics 131(3): 543–556.

[bibr21-0018726718754992] Derks B Van Laar C Ellemers N (2016) The Queen Bee phenomenon: Why women leaders distance themselves from junior women. Leadership Quarterly 27(3): 456–469.

[bibr22-0018726718754992] DiBenigno J Kellogg KC (2014) Beyond occupational differences: The importance of cross-cutting demographics and dyadic toolkits for collaboration in a U.S. Hospital. Administrative Science Quarterly 59(3): 375–408.

[bibr23-0018726718754992] Dillon RS (2007) Respect: A philosophical perspective. Gruppendynamik Und Organisations-beratung 38(2): 201–212.

[bibr24-0018726718754992] DIW (2015) DIW–German Institute for Economic Research. DIW poll 2015. Available at: https://www.diw.de/en/diw_01.c.495406.en/topics_news/women_executive_barometer_2015_highest_decision_making_bodies_in_german_companies_still_male_dominated.html (accessed 5 March 2018).

[bibr25-0018726718754992] Douglas C (2012) The moderating role of leader and follower sex in dyads on the leadership behavior–leader effectiveness relationships. The Leadership Quarterly 23(1): 163–175.

[bibr26-0018726718754992] Duffy MK Ferrier WJ (2003) Birds of a feather…? How supervisor-subordinate dissimilarity moderates the influence of supervisor behaviors on workplace attitudes. Group & Organization Management 28(2): 217–248.

[bibr27-0018726718754992] Eagly AH (2005) Achieving relational authenticity in leadership: Does gender matter? Leadership Quarterly 16(3): 459–474.

[bibr28-0018726718754992] Eagly AH (2007) Female leadership advantage and disadvantage: Resolving the contradictions. Psychology of Women Quarterly 31(1): 1–12.

[bibr29-0018726718754992] Eagly AH Carli LL (2003) The female leadership advantage: An evaluation of the evidence. Leadership Quarterly 14(6): 807–834.

[bibr30-0018726718754992] Eagly AH Karau SJ (2002) Role congruity theory of prejudice toward female leaders. Psychological Review 109(3): 573–598. 12088246 10.1037/0033-295x.109.3.573

[bibr31-0018726718754992] Eagly AH Wood W (1991) Explaining sex differences in social behavior: A meta-analytic perspective. Personality and Social Psychology Bulletin 17(3): 306–315.

[bibr32-0018726718754992] Eagly AH Karau SJ Makhijani MG (1995) Gender and the effectiveness of leaders: A meta-analysis. Psychological Bulletin 117(1): 125–145. 7870858 10.1037/0033-2909.117.1.125

[bibr33-0018726718754992] Eagly AH Makhijani MG Klonsky BG (1992) Gender and the evaluation of leaders: A meta-analysis. Psychological Bulletin 111(1): 3–22. 10.1037/0033-2909.117.1.125 7870858

[bibr34-0018726718754992] Elsesser KM Lever J (2011) Does gender bias against female leaders persist? Quantitative and qualitative data from a large-scale survey. Human Relations 64(12): 1555–1578.

[bibr35-0018726718754992] Fiske ST Cuddy AJC Glick P Xu J (2002) A model of (often mixed) stereotype content: Competence and warmth respectively follow from perceived status and competition. Journal of Personality and Social Psychology 82(6): 878–902. 12051578

[bibr36-0018726718754992] Gallup (2015) Gallup poll social series: Work and education. Gallup poll 2015. Available at: http://news.gallup.com/businessjournal/183026/female-bosses-engaging-male-bosses.aspx (accessed 5 March 2018).

[bibr37-0018726718754992] Genovese M (1993) Women as National Leaders. Newbury Park, CA: SAGE.

[bibr38-0018726718754992] Gerstner CR Day DV (1997) Meta-analytic review of leader-member exchange theory: Correlates and construct issues. Journal of Applied Psychology 82(6): 827–844.

[bibr39-0018726718754992] Giessner SR Van Quaquebeke N (2010) Using a relational models perspective to understand normatively appropriate conduct in ethical leadership. Journal of Business Ethics 95(S1): 43–55.

[bibr40-0018726718754992] Giessner SR van Knippenberg D Sleebos E (2009) License to fail? How leader group prototypicality moderates the effects of leader performance on perceptions of leadership effectiveness. Leadership Quarterly 20(3): 434–451.

[bibr41-0018726718754992] Grover SL (2013) Unraveling respect in organization studies. Human Relations 67(1): 27–51.

[bibr42-0018726718754992] Guillaume YRF Brodbeck FC Riketta M (2012) Surface- and deep-level dissimilarity effects on social integration and individual effectiveness related outcomes in work groups: A meta-analytic integration. Journal of Occupational and Organizational Psychology 85(1): 80–115.

[bibr43-0018726718754992] Hall RJ Lord RG (1995) Multi-level information-processing explanations of followers’ leadership perceptions. Leadership Quarterly 6(3): 265–287.

[bibr44-0018726718754992] Haslam SA Ryan MK (2008) The road to the glass cliff: Differences in the perceived suitability of men and women for leadership positions in succeeding and failing organizations. Leadership Quarterly 19(5): 530–546.

[bibr45-0018726718754992] Heilman ME (2001) Description and prescription: How gender stereotypes prevent women’s ascent up the organizational ladder. Journal of Social Issues 57(4): 657–674.

[bibr46-0018726718754992] Hekman DR Johnson S Foo MD Yang W (2017) Does diversity-valuing behavior result in diminished performance ratings for nonwhite and female leaders? Academy of Management Journal 60(2): 771–797.

[bibr47-0018726718754992] Hofstede G (1983) National cultures in four dimensions: A research-based theory of cultural differences among nations. International Studies of Management & Organization 13(1): 46–74.

[bibr48-0018726718754992] Hogg MA Hains SC Mason I (1998) Identification and leadership in small groups: Salience, frame of reference, and leader stereotypicality effects on leader evaluations. Journal of Personality and Social Psychology 75(5): 1248–1263.

[bibr49-0018726718754992] Huo YJ Binning KR (2008) Why the Psychological Experience of Respect Matters in Group Life: An Integrative Account. Social and Personality Psychology Compass 2(4): 1570–1585.

[bibr50-0018726718754992] Huo YJ Binning KR Molina LE (2010) Testing an Integrative Model of Respect: Implications for Social Engagement and Well-Being. Personality and Social Psychology Bulletin 36(2): 200–212. 20032268 10.1177/0146167209356787

[bibr51-0018726718754992] Ilies R Nahrgang JD Morgeson FP (2007) Leader-member exchange and citizenship behaviors: A meta-analysis. Journal of Applied Psychology 92(1): 269–277. 17227168 10.1037/0021-9010.92.1.269

[bibr52-0018726718754992] Johnson SK Murphy SE Zewdie S Reichard RJ (2008) The strong, sensitive type: Effects of gender stereotypes and leadership prototypes on the evaluation of male and female leaders. Organizational Behavior and Human Decision Processes 106(1): 39–60.

[bibr53-0018726718754992] Kark R Waismel-Manor R Shamir B (2012) Does valuing androgyny and femininity lead to a female advantage? The relationship between gender-role, transformational leadership and identification. The Leadership Quarterly 23(3): 620–640.

[bibr54-0018726718754992] Koenig AM Eagly AH (2014) Evidence for the social role theory of stereotype content: Observations of groups’ roles shape stereotypes. Journal of Personality and Social Psychology Social Psychology 107(3): 371–392. 10.1037/a0037215 25133722

[bibr55-0018726718754992] Koenig AM Eagly AH Mitchell AA Ristikari T (2011) Are leader stereotypes masculine? A meta-analysis of three research paradigms. Psychological Bulletin 137(4): 616–642. 21639606 10.1037/a0023557

[bibr56-0018726718754992] Landau J (1995) The relationship of race and gender to managers’ ratings of promotion potential. Journal of Organizational Behavior 16(4): 391–400.

[bibr57-0018726718754992] Lankau MJ Riordan CM Thomas CH (2005) The effects of similarity and liking in formal relationships between mentors and protégés. Journal of Vocational Behavior 67(2): 252–265.

[bibr58-0018726718754992] Lincoln JR Miller J (1979) Work and friendship ties in organizations: A comparative analysis of relation networks. Administrative Science Quarterly 24(2): 181–199.

[bibr59-0018726718754992] Lord RG Maher KJ (2002) Leadership and Information Processing: Linking Perceptions and Performance. New York: Routledge.

[bibr60-0018726718754992] Luksyte A Avery DR (2015) Exploring burnout and work-family facilitation as factors influencing why and when relational demography diminishes employee citizenship. Journal of Occupational and Organizational Psychology 88(4): 750–772.

[bibr61-0018726718754992] McAllister DJ (1995) Affect- and cognition-based trust as foundations for interpersonal cooperation in organizations. Academy of Management Journal 38(1): 24–59.

[bibr62-0018726718754992] Morrison AM Von Glinow MA (1990) Women and minorities in management. American Psychologist 45(2): 200–208.

[bibr63-0018726718754992] Netchaeva E Kouchaki M Sheppard LD (2015) A man’s (precarious) place: Men’s experienced threat and self-assertive reactions to female superiors. Personality and Social Psychology Bulletin 41(9): 1247–1259. 26162611 10.1177/0146167215593491

[bibr64-0018726718754992] Ng TWH Eby LT Sorensen KL Feldman DC (2005) Predictors of objective and subjective career success: A meta-analysis. Personnel Psychology 58(2): 367–408.

[bibr65-0018726718754992] O’Reilly CA Caldwell DF Barnett WP (1989) Work group demography, social integration, and turnover. Administrative Science Quarterly 34(1): 21–37.

[bibr66-0018726718754992] Paustian-Underdahl SC Walker LS Woehr DJ (2014) Gender and perceptions of leadership effectiveness: A meta-analysis of contextual moderators. The Journal of Applied Psychology 99(6): 1129–1145. 24773399 10.1037/a0036751

[bibr67-0018726718754992] Pearce CL (2007) The future of leadership development: The importance of identity, multi-level approaches, self-leadership, physical fitness, shared leadership, networking, creativity, emotions, spirituality and on-boarding processes. Human Resource Management Review 17(4): 355–359.

[bibr68-0018726718754992] Pelled LH Xin KR (1997) Birds of a feather: Leader-member demographic similarity and organizational attachment in Mexico. Leadership Quarterly 8(4): 433–450.

[bibr69-0018726718754992] Phillips KW Rothbard NP Dumas TL (2009) To disclose or not to disclose? Status distance and self-disclosure in diverse environments. Academy of Management Review 34(4): 710–732.

[bibr70-0018726718754992] Podsakoff PM Mackenzie SB Lee J-Y Podsakoff NP (2003) Common method biases in behavioral research: A critical review of the literature and recommended remedies. Journal of Applied Psychology 88(5): 879–903. 14516251 10.1037/0021-9010.88.5.879

[bibr71-0018726718754992] Powell GN Butterfield DA (2015) The preference to work for a man or a woman: A matter of sex and gender? Journal of Vocational Behavior 86: 28–37.

[bibr72-0018726718754992] Renger D Simon B (2011) Social recognition as an equal: The role of equality-based respect in group life. European Journal of Social Psychology 41(4): 501–507.

[bibr73-0018726718754992] Rink F Ryan MK Stoker JI (2013) Social resources at a time of crisis: How gender stereotypes inform gendered leader evaluations. European Journal of Social Psychology 43(5): 381–392.

[bibr74-0018726718754992] Rogers KM Ashforth BE (2014) Respect in organizations: Feeling valued as “we” and “me”. Journal of Management 43(5): 1578–1608.

[bibr75-0018726718754992] Sarbin T Allen V (1954) Role theory. In: Lindzey G Aronson E (eds) The Handbook of Social Psychology. Reading: Addison-Wesley, 223–258.

[bibr76-0018726718754992] Schein VE (1976) Think manager-think male. Atlanta Economic Review 26(2): 21–24.

[bibr77-0018726718754992] Schein VE Mueller R Lituchy T Liu J (1996) Think manager–think male: A global phenomenon? Journal of Organizational Behavior 17(1): 33–41.

[bibr78-0018726718754992] Schuh SC Hernandez Bark AS Van Quaquebeke N et al (2013) Gender differences in leadership role occupancy: The mediating role of powermotivation. Journal of Business Ethics 120(3): 363–379.

[bibr79-0018726718754992] Scott KA Brown DJ (2006) Female first, leader second? Gender bias in the encoding of leadership behavior. Organizational Behavior and Human Decision Processes 101(2): 230–242.

[bibr80-0018726718754992] Sczesny S (2003) A closer look beneath the surface: Various facets of the think-manager–think-male stereotype. Sex Roles 49(7/8): 353–363.

[bibr81-0018726718754992] Simon B Lücken M Stürmer S (2006) The added value of respect: Reaching across inequality. British Journal of Social Psychology 45(3): 535–546. 16984719 10.1348/014466605X57637

[bibr82-0018726718754992] Sluss DM Ashforth BE (2008) How relational and organizational identification converge: Processes and conditions. Organization Science 19(6): 807–823.

[bibr83-0018726718754992] Smith HJ Tyler TR Huo YJ et al (1998) The self-relevant implications of the group-value model: Group membership, self-worth, and treatment quality. Journal of Experimental Social Psychology 34(5): 470–493.

[bibr84-0018726718754992] Spector PE Brannick MT (2011) Methodological urban legends: The misuse of statistical control wariables. Organizational Research Methods 14(2): 287–305.

[bibr85-0018726718754992] Stoker JI Van der Velde M Lammers J (2012) Factors relating to managerial stereotypes: The role of gender of the employee and the manager and management gender ratio. Journal of Business and Psychology 27(1): 31–42. 22363099 10.1007/s10869-011-9210-0 PMC3278615

[bibr86-0018726718754992] Strauss JP Barrick MR Connerley ML (2001) An investigation of personality similarity effects (relational and perceived) on peer and supervisor ratings and the role of familiarity and liking. Journal of Occupational and Organizational Psychology 74(5): 637–657.

[bibr87-0018726718754992] Triana MDC Richard OC Yücel İ (2016) Status incongruence and supervisor gender as moderators of the transformational leadership to subordinate affective organizational commitment relationship. Personnel Psychology 70(2): 429–467.

[bibr88-0018726718754992] Tsui AS O’Reilly CA (1989) Beyond simple demographic effects: The importance of relational demography in superior-subordinate dyads. Academy of Management Journal 32(2): 402–423.

[bibr89-0018726718754992] Tsui AS Egan TD O’Reilly CA (1992) Being different: Relational demography and organizational attachment. Administrative Science Quarterly 37(4): 549–579.

[bibr90-0018726718754992] Tsui AS Porter LW Egan TD et al (2002) When both similarities and dissimilarities matter: Extending the concept of relational demography. Human Relations 55(8): 899–929.

[bibr91-0018726718754992] Turban DB Dougherty TW Lee FK (2002) Gender, race, and perceived similarity effects in developmental relationships: The moderating role of relationship duration. Journal of Vocational Behavior 61(2): 240–262.

[bibr92-0018726718754992] Turner N Hogg MA Oakes PJ et al (1987) Rediscovering the Social Group: A Self-Categorization Theory. Oxford: Blackwell.

[bibr93-0018726718754992] Tyler TR (2001) Public trust and confidence in legal authorities: What do majority and minority group members want from the law and legal institutions? Behavioral Sciences & the Law 19(2): 215–235. 11385699 10.1002/bsl.438

[bibr94-0018726718754992] Tyler TR Smith HJ (1999) Justice, social identity and group processes. In: Tyler TR Kramer RM John OP (eds) The Psychology of the Social Self. Mahaw, NJ: Erlbaum, 223–264.

[bibr95-0018726718754992] Van Engen ML Willemsen TM (2004) Sex and leadership styles: A meta-analysis of research published in the 1990s. Psychological Reports 94(1): 3–18. 15077742 10.2466/pr0.94.1.3-18

[bibr96-0018726718754992] van Knippenberg D Hogg MA (2003) A social identity model of leadership effectiveness in organizations. In: Kramer RM Staw BM (eds) Research in Organizational Behavior, Vol. 25 Amsterdam: Elsevier, 243–295.

[bibr97-0018726718754992] van Knippenberg D Sitkin SB (2013) A critical assessment of charismatic—transformational leadership research: Back to the drawing board? The Academy of Management Annals 7(1): 1–60.

[bibr98-0018726718754992] Van Quaquebeke N Eckloff T (2010) Defining respectful leadership: What it is, how it can be measured, and another glimpse at what it is related to. Journal of Business Ethics 91(3): 343–358.

[bibr99-0018726718754992] Van Quaquebeke N Zenker S Eckloff T (2009) Find out how much it means to me! The importance of interpersonal respect in work values compared to perceived organizational practices. Journal of Business Ethics 89(3): 423–431.

[bibr100-0018726718754992] Vecchio RP Boatwright KJ (2002) Preferences for idealized styles of supervision. The Leadership Quarterly 13(4): 327–342.

[bibr101-0018726718754992] Vial AC Napier JL Brescoll VL (2016) A bed of thorns: Female leaders and the self-reinforcing cycle of illegitimacy. The Leadership Quarterly 27(3): 400–414.

[bibr102-0018726718754992] Wang A-C Chiang JT-J Tsai C-Y et al (2013) Gender makes the difference: The moderating role of leader gender on the relationship between leadership styles and subordinate performance. Organizational Behavior and Human Decision Processes 122(2): 101–113.

[bibr103-0018726718754992] Wesolowski MA Mossholder KW (1997) Relational demography in supervisor subordinate dyads: Impact on subordinate job satisfaction, burnout, and perceived procedural justice. Journal of Organizational Behavior 18(4): 351–362.

[bibr104-0018726718754992] West SG Aiken LS Krull JL (1996) Experimental personality designs: Analyzing categorical by continuous variable interactions. Journal of Personality 64(1): 1–48. 8656311 10.1111/j.1467-6494.1996.tb00813.x

